# Fine Mapping of a Major Backfat QTL Reveals a Causal Regulatory Variant Affecting the *CCND2* Gene

**DOI:** 10.3389/fgene.2022.871516

**Published:** 2022-05-25

**Authors:** Haniel C. Oliveira, Martijn F. L. Derks, Marcos S. Lopes, Ole Madsen, Barbara Harlizius, Maren van Son, Eli H. Grindflek, Marta Gòdia, Arne B. Gjuvsland, Pamela Itajara Otto, Martien A. M. Groenen, Simone E. F. Guimaraes

**Affiliations:** ^1^ Department of Animal Science, Universidade Federal de Viçosa, Viçosa, Brazil; ^2^ Topigs Norsvin Research Center, Beuningen, Netherlands; ^3^ Animal Breeding and Genomics, Wageningen University & Research, Wageningen, Netherlands; ^4^ Topigs Norsvin, Curitiba, Brazil; ^5^ Norsvin SA, Hamar, Norway; ^6^ Department of Animal Science, Universidade Federal de Santa Maria, Santa Maria, Brazil

**Keywords:** GWAS, pig genomics, animal breeding, finemapping, backfat

## Abstract

Backfat is an important trait in pork production, and it has been included in the breeding objectives of genetic companies for decades. Although adipose tissue is a good energy storage, excessive fat results in reduced efficiency and economical losses. A large QTL for backfat thickness on chromosome 5 is still segregating in different commercial pig breeds. We fine mapped this QTL region using a genome-wide association analysis (GWAS) with 133,358 genotyped animals from five commercial populations (Landrace, Pietrain, Large White, Synthetic, and Duroc) imputed to the porcine 660K SNP chip. The lead SNP was located at 5:66103958 (G/A) within the third intron of the *CCND2* gene, with the G allele associated with more backfat, while the A allele is associated with less backfat. We further phased the QTL region to discover a core haplotype of five SNPs associated with low backfat across three breeds. Linkage disequilibrium analysis using whole-genome sequence data revealed three candidate causal variants within intronic regions and downstream of the *CCND2* gene, including the lead SNP. We evaluated the association of the lead SNP with the expression of the genes in the QTL region (including *CCND2*) in a large cohort of 100 crossbred samples, sequenced in four different tissues (lung, spleen, liver, muscle). Results show that the A allele increases the expression of *CCND2* in an additive way in three out of four tissues. Our findings indicate that the causal variant for this QTL region is a regulatory variant within the third intron of the *CCND2* gene affecting the expression of *CCND2*.

## Introduction

Backfat (BF) is an important trait in pork production included in the breeding objectives of genetic companies for decades (Merks, 2000). Pig commercial lines have been selected for efficient meat production selecting for growth and leanness traits and thereby reducing backfat. However, a strong selection for decreased backfat also decreases other fat-related traits such as intra-muscular fat, which is highly valuable for meat quality (Lonergan et al., 2001; Fontanesi et al., 2012; Chen et al., 2014).

Adipose tissue has an important role as the storage of energy (Wang et al., 2016), however, excessive fat may cause economic implications in pig breeding affecting growth, feed efficiency, and meat quality (Lonergan et al., 2001). Furthermore, fat and fatty acids contribute significantly to the quality and nutritional content of the meat (Wood et al., 2008). The amounts of saturated fatty acids (SFA), monounsaturated fatty acids (MUFA) and polyunsaturated fatty acids (PUFA) are important for the meat processing industry.

Hence, to better understand quantitative traits such as backfat, further insight in the genetic basis of these traits is required. The development of commercial SNP chips has enabled genome-wide association studies (GWAS) to efficiently map regions throughout the genome affecting fat-related traits (Fontanesi et al., 2012; Fowler et al., 2013; Okumura et al., 2013; Do et al., 2014; Gozalo-Marcilla et al., 2021). While GWAS identify significant marker associations, the current commercial SNP chip density often leads to clusters of markers (due to extended linkage disequilibrium) covering a region that is still too large to allow accurate identification of the responsible gene(s) or variant(s). Hence, the need for further fine mapping is required to find causative relations between gene(s) or variants that affect economically-important traits such as backfat. The detection and integration of expression quantitative trait loci (eQTLs) can facilitate further fine mapping of QTL regions to study the genetic architecture of complex traits. The eQTL analysis allows to identify variation associated with changes in gene expression that underly the phenotypic differences observed in complex traits.

One region on chromosome 5 (66 Mb) has shown strong association with backfat in a wide range of populations. Gozalo-Marcilla et al. 2021 proposed the *FGF23* gene as likely causal given the association with phosphate homeostasis. In this study, we investigate the same QTL region on chromosome 5 with strong association with backfat across four commercial pig populations. Further, we aimed to fine map this QTL region to identify causative mutation(s) related to backfat and to better understand the biology of this trait. We concluded that the causative variant of this QTL region is a regulatory SNP in the third intron of the *CCND2* gene.

## Material and Methods

### Phenotypic Data

The data consisted of five commercial pig populations; Synthetic (Large-White based), Pietrain, Landrace, Large-White and Duroc **(** Table 1), obtained from herds owned by Topigs Norsvin. The phenotypic data was composed by backfat records, measured at the end of the test period (average live weight of 120 kg) in each evaluated population. The data were classified in two datasets: PHENOTYPED, which consisted of all phenotyped animals and their contemporary’s animals; and GENOTYPED, which is a subset of phenotyped animals with genotypes and phenotypes (Table 1).

**TABLE 1:** Summary statistics of the backfat (millimeters) per datasets and per evaluated population.

Population	Dataset	Number	Mean	SD	Min	Max
Synthetic	PHENOTYPED^a^	125,319	9.98	2.28	3.5	29.4
	GENOTYPED^b^	31,183	9.48	1.97	3.5	23.5
Pietrain	PHENOTYPED	97,358	7.72	1.58	3.5	28.8
	GENOTYPED	16,189	7.32	1.38	3.5	19.9
Landrace	PHENOTYPED	223,723	7.01	1.66	3.5	27.0
	GENOTYPED	38,298	7.59	1.68	3.9	20.8
Large-White	PHENOTYPED	175,757	12.62	2.74	3.5	29.9
	GENOTYPED	36,414	12.52	2.6	4.0	29.5
Duroc	PHENOTYPED	37,991	8.70	2.04	4.0	20.0
	GENOTYPED	11,274	8.13	2.19	4.0	20.0

SD, standard deviation; Min, minimum value; Max, maximum value.

a Phenotyped: consisted of all phenotyped animals and their contemporaries, used for pre-correction of the phenotypes.

b Genotyped: subset that includes only genotyped and phenotyped animals, used in the genome-wide association analyses.

Using PHENOTYPED dataset, phenotypes were pre-corrected for all non-genetic effects to use as response variable in further analysis. For that, the non-genetic effects were estimated within population using the following animal linear model in ASReml v3.0 (Gilmour et al., 2009):

$${\text{BF}}_{\text{ijkl}}=\text{μ}+{\text{sex}}_{\text{i}}+{\text{hym}}_{\text{j}}+{\text{a}}_{\text{k}}+{\text{litter}}_{\text{l}}+{\text{e}}_{\text{ijkl}}$$ (1)

where ${\text{BF}}_{\text{ijkl}}$ is the phenotypic observations (BF) of the *k* animal; μ is the overall mean; ${\text{sex}}_{\text{i}}$ is the fixed effect of sex *i*; ${\text{hym}}_{\text{j}}$ is the fixed effect of the herd-year-month *j* of birth; ${\text{a}}_{\text{k}}$ is the random additive genetic effect of animal *k*; ${\text{litter}}_{\text{l}}$ is the random effect of litter *l* and ${\text{e}}_{\text{ijkl}}$ is the random residual effect. It was assumed that a ∼*N* (**0**, **A** ${\text{σ}}_{\text{a}}^{2}$ ), l ∼*N* (**0**, **I** ${\text{σ}}_{\text{l}}^{2}$ ) and e ∼*N* (**0**, **I** ${\text{σ}}_{\text{e}}^{2}$ ), where ${\text{σ}}_{\text{a}}^{2}$ , ${\text{σ}}_{\text{l}}^{2}$ and ${\text{σ}}_{\text{e}}^{2}$ are the additive genetic variance, litter variance and residual variance, respectively; **A** is a pedigree-based numerator relationship matrix and **I** is the identity matrix.

### Genotyping and Quality Control

In total, 134,887 animals were genotyped across the different populations and SNP chip densities (Table 2). The GENOTYPED dataset includes animals genotyped using different Illumina’s medium density SNP chips (50, 60 and 80K), as well as animals genotyped with the high-density Axiom™ Porcine Genotyping Array 660K (Thermo Fisher Scientific, Waltham, Massachusetts, United States) (Table 2).

**TABLE 2:** Number of genotyped and sequenced animals available with backfat measurements per population and per SNP chip.

Population	Illumina 50K	Illumina 60K	Illumina 80K	Axiom™ 660K	Total genotyped	WGS
Synthetic	25,193	739	5,251	319	31,502	187
Pietrain	10,396	1,414	4,379	228	16,417	40
Landrace	38,298	-	-	441	38,739	227
Large-White	36,414	-	-	401	36,815	205
Duroc	6,139	1,174	3,961	140	11,414	-
Total	116,440	3,327	13,591	1,529	134,887	

Genotype quality control analysis was performed within population and SNP chip to exclude SNPs with genotype call rate <0.95, minor allele frequency <0.01, strong deviation from Hardy-Weinberg equilibrium *p*-value < 1 × 10^-12^, SNPs located on sex chromosomes and unmapped SNPs. The positions of the SNPs are based on the Sscrofa11.1 assembly of the reference genome (Warr et al., 2020a). All genotyped animals had a frequency of missing genotypes <0.05 and were therefore all kept for further analyses.

After the quality control, the medium density genotypes were imputed towards the high density genotypes within population using Fimpute v2.2 (Sargolzaei et al., 2014). After imputation, a total of 31,183; 16,189; 38,298; 36,414 and 11,274 animals and 488,585, 504,525, 443,619, 518,659 and 417,532 SNPs were available for the Synthetic, Pietrain, Landrace, Large-White and Duroc populations, respectively, and used for the GWAS.

### Genome-Wide Association Analysis

A single-SNP GWAS was performed with the imputed GENOTYPED dataset for each population using the following linear animal model in GCTA software (Yang et al., 2011, 2014):

$$\text{y}{\text{∗}}_{\text{k}}=\mu +\text{X}\hat{\beta }+u_{\text{k}}+{\text{e}}_{\text{k}}$$ (2)

where y∗k is the pre-corrected phenotype of the *k* animal (pre-corrected for all non-genetic effects); μ is the average of the pre-corrected phenotype; *X* is the genotype, coded as 0, 1, or 2 copies of one of the alleles of the *k* animal for the evaluated SNP; $\hat{\beta }$ is the unknown allele substitution effect of the evaluated SNP; u_k_ is the residual polygenic effect, assuming u ∼N (**0**, **G** ${\text{σ}}_{\text{u}}^{2}$ ), which accounted for the (co)variances between animals due to relationships by formation of an **G** matrix (genomic numerator relationship matrix build using the imputed genotypes), ${\text{σ}}_{\text{u}}^{2}$ is the additive genetic variance; and ${\text{e}}_{\text{k}}$ is the random residual effect which was assumed to be distributed as ∼N (**0**, **I** ${\text{σ}}_{\text{e}}^{2}$ ).

The proportion of phenotypic variance explained by a SNP was defined as $\frac{\sigma_{SNP}^{2}}{\sigma_{P}^{2}}$ , where $\sigma_{P}^{2}$ is total phenotypic variance (sum of the additive and residual variances) which was estimated based on model (2) without a SNP effect, and $\sigma_{SNP}^{2}=2pq{\left(\beta \right)}^{2}$ , where p and q are the allele frequencies and $\hat{\beta }$ the estimated allele substitution effect of the evaluated SNP. Variance components were also estimated using model (2) without a SNP effect and the heritability was defined as the proportion of $\sigma_{P}^{2}$ explained by the genetic variance. The association between a SNP and the phenotype was declared significant when–log_10_(*p*-value) > 8.

### Fine-Mapping

To search for possible causal mutations at the chromosome 5 backfat locus, fine mapping analyses using high-throughput sequencing (WGS, ChIP-Seq, ATAC-Seq and RNA-Seq data) was performed using following steps: (1) building and calculation of haplotype frequencies; (2) sequence data processing and evaluation of variants within the haplotype region; (3) evaluation of protein interactions and gene expression.

### Haplotype Analysis

From the GWAS results, the most significant SNP (lead SNP) was identified. Subsequently we performed a haplotype analysis up-and downstream of the lead SNP. For that, firstly the Beagle v5.1 software (Browning et al., 2018) was used to phase the imputed genotypes taking 0.5 Mb downstream and 0.5 Mb upstream of the lead SNP (SSC5:66,103,958bp) in the QTL region SSC5:66 ± 0.5 Mb. The lead SNP and the 40 markers with closest vicinity of the lead SNP (20 markers on each side) were selected and used in the HaploPi pipeline (Oliveira, 2019) to build and calculate the haplotype frequencies. After performing the haplotype analyses, the core haplotype (SSC5: 66094630-66140348) was used to perform a haplotype-based association analysis using the number of copies of the core haplotype. For that, the same mixed-model described for the single-SNP GWAS (model 2) was used, in which the SNP genotypes were replaced by the genotype of the core haplotype (0, 1 or 2).

### Sequence Data

Whole-genome sequence (WGS) data was used to access the complete set of variants within the haplotype region (SSC5:66094630-66140348). For this purpose, 659 animals (Synthetic = 187; Pietrain = 40; Landrace = 227; Large White = 205) were available (Table 2). Genomic DNA from ear biopsies was extracted using BioSprint DNA Kit (Qiagen) and its concentration and quality was measured using NanoDrop ND-1000 spectrophotometer (Thermo Fisher Scientific). The data was sequenced on Illumina Hiseq 2000 150 bp paired-end reads. The reads were aligned to the Sscrofa11.1 (Warr et al., 2020b) using BWA-MEM v0.7.15 (Li and Durbin, 2009) with an average mappability of 96.51% and a sample coverage ranging from 6.6–22.7X (∼10X average). SAMtools v1.9 dedup function was used to remove PCR duplicates (Li et al., 2009). Variant calling was performed with Freebayes v1.1.0 with following settings: min-base-quality 10, min-alternate-fraction 0.2, haplotype-length 0 and min-alternate-count 2 (Garrison and Marth, 2012). Variants with Phred quality score <20, and within 3 bp of an indel were discarded (Li et al., 2009). Variants were annotated using the Ensembl variant effect predictor (VEP, release 99) (McLaren et al., 2016). The impact of missense variants was predicted using the SIFT tool v6.2.1 (Kumar et al., 2009).

### ChIP-Seq and ATAC-Seq Analysis

Chromatin immunoprecipitation–coupled sequencing (ChIP-Seq) marked by trimethylated histone H3 lysine 4 (H3K4me3), mono-methylation histone H3 lysine 4 (H3K4me1) and acetylated histone 3 lysine 27(H3K27ac) and ATAC-Seq data were downloaded from the European Nucleotide Archive (ENA) with accession numbers PRJEB28147 (ChIP-Seq - brain, liver, muscle and testis), and PRJNA665194 (ATAC-Seq – adipose, cerebellum, cerebral cortex, hypothalamus, liver, lung, skeletal muscle and spleen). ChIP-Seq marks and ATAC-Seq data were aligned against the pig reference genome (Sscrofa 11.1) using BWA-mem v. 0.7.17 (Li, 2013), pre-processed using SAMtools v. 1.13 (Li and Durbin, 2009) and broad and narrow peaks calling was performed on MACS v2.2.6 (Zhang et al., 2008) (parameters used for mapping, pre-processing and peak calling can be found in Supplementary Material S1.

### RNA-Sequencing in Crossbred Animals in Four Tissues for eQTL Analysis

Four different tissues: liver, spleen, lung and muscle were subjected to RNA extraction from 100 animals. The animals were F2 crosses resulting from a F1 sow (Landrace*LargeWhite) and a Synthetic boar line. The collected tissue was stored with RNAlater (Thermo Fisher Scientific) at -80°C until further use. RNA was extracted from the 400 samples using the QIAshredder homogenizer kit (Qiagen) and RNA extraction was performed with the Rneasy kit (Qiagen) following manufacture’s guidelines. Once all the samples passed the required quality control parameters, they were sequenced at 150 bp paired-end in an Illumina 6000 sequencing platform. DNA from 87 of these animals was also extracted from the spleen tissue and was subjected to high density genotyping with the Axiom™ Porcine Genotyping Array (Thermo Fisher Scientific) that queries 660K variant markers.

For the bioinformatic analysis, RNA-Seq reads were trimmed using Trim Galore v0.3.7 (https://www.bioinformatics.babraham.ac.uk/projects/trim_galore/). We mapped the RNA-seq data to the Sscrofa11.1 reference genome and Ensembl version 104 using STAR v2.7.8 (Dobin et al., 2013). Alignments with an alignment MAPQ score <30 were filtered using SAMtools v0.1.19 (Li and Durbin, 2009). Gene counts were determined using htseq-count v0.11.1, and then TMM-normalized as counts per million (CPM) with EdgeR (Robinson et al., 2009). Plotting of expression levels per genotype class was performed using the seaborn python package (Waskom, 2021). For the eQTL analysis, genotypes were firstly filtered as in (Derks et al., 2021) with plink v.1.9 (Purcell et al., 2007). Only genes ±1 Mbp from the target lead SNP of interest were used for the analysis. The single-SNP association analysis was carried for each of the tissues with the GCTA v1.25.3 software (Yang et al., 2011) with the following model:

$$Y_{i}=µ+SNP_{i}+e_{i}$$ (3)

where ( $Y_{i}$ ) is the CPM gene abundance modeled as a function of the population mean (µ), fixed effect of each SNP ( $SNP_{i}$ ), and a random residual effect ( $e_{i}$ ). The genetic variance explained by a SNP ( $\sigma_{SNP}^{2}$ = *2pqα* ^*2*^) was estimated based on the allele frequencies (*p* and *q*) and the estimated allele substitution effect (*α*). The proportion of phenotypic variance explained by the SNP was defined as $\sigma_{SNP}^{2}/\sigma_{P}^{2}$ , where $\sigma_{P}^{2}$ is total phenotypic variance (sum of the additive and residual variances) which was estimated based on model (3) without a SNP effect. Significant association between a SNP and expression was detected using a *p*-value < 0.05.

## Results

The heritability, defined as the proportion of the total phenotypic variance explained by additive genetic variance, for backfat ranged from 0.42 to 0.54 among the five evaluated pig populations: Synthetic (0.54 ± 0.01), Pietrain (0.50 ± 0.01), Landrace (0.42 ± 0.01), Large White (0.52 ± 0.01) and Duroc (0.43 ± 0.01) (Table 3).

**TABLE 3:** Summary of the genetic parameters for backfat evaluated in five pig populations.

Population	$\sigma_{a}^{2}$	$\sigma_{e}^{2}$	$\sigma_{p}^{2}$	h^2^
Synthetic	1.19 ± 0.04	1.01 ± 0.01	2.21 ± 0.04	0.54 ± 0.01
Pietrain	0.56 ± 0.02	0.57 ± 0.01	1.13 ± 0.02	0.50 ± 0.01
Landrace	0.55 ± 0.02	0.77 ± 0.01	1.32 ± 0.02	0.42 ± 0.01
Large-White	1.68 ± 0.05	1.56 ± 0.01	3.24 ± 0.05	0.52 ± 0.01
Duroc	0.78 ± 0.04	1.03 ± 0.02	1.82 ± 0.04	0.43 ± 0.01

$\sigma_{a}^{2}$ , Variance additive; $\sigma_{e}^{2}$ , Variance environment; $\sigma_{p}^{2}$ , Variance phenotypic; h2, Heritability.

### Genome-Wide Association Study

**FIGURE 1:**
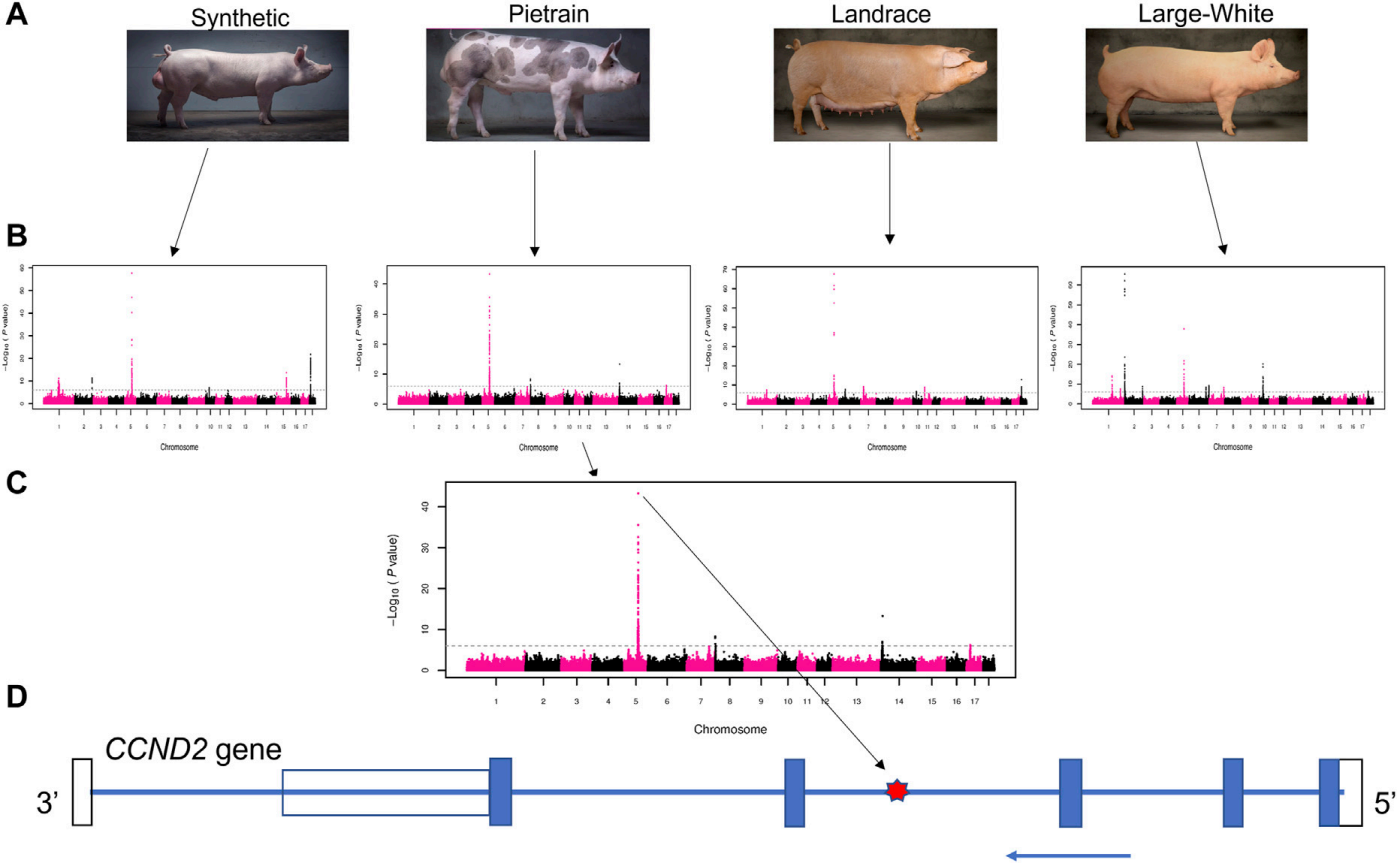
Schematic representation of the GWAS results. **(A)** Four pig populations that presented the significant overlapping GWAS peaks on chromosome 5: Synthetic (Large-White based), Pietrain, Landrace and Large-White. **(B)** GWAS results for backfat in four populations. **(C)** Manhattan plot showing the most significant SNP (lead SNP - rs80985094) in the Pietrain population. **(D)** *CCND2* gene model. The location of the lead SNP in the third intron is indicated with a red star. The blue arrow indicates the coding strand, blue boxes depict exons, the open bars the untranslated regions.

In this study, the use of large-scale genotype data allowed us to identify a A/G SNP (rs80985094: 5:66,103,958) on SSC5 with a strong association with backfat across populations. This SNP showed significant association with backfat in four (Synthetic, Pietrain, Landrace and Large White) out of the five evaluated pig populations (Figure 1). The association between rs80985094 and backfat was not identified in the Duroc population because the G allele, associated with increased backfat is fixed.

The most significant SNP (rs80985094) showed a -log10 (*p*-value) of 57.67, 43.29, 67.61 and 37.91 in the Synthetic, Pietrain, Landrace and Large-White population, respectively. The lead SNP, located at 66,103,958 bp on SSC5, explains up to 6% of the genetic variance in backfat (Table 4). Among the four populations which showed significant association between the lead SNP and backfat, the frequency of the allele associated with increased backfat (G) is the highest in Large-White population (0.79). In the other populations, the G allele is the minor allele (Table 4).

**TABLE 4:** Description of the lead SNP (rs80985094) for backfat for each population.

Population	F(G)	-log10 (p-value)	β	SE	$\sigma_{a}^{2}$ Exp	$\sigma_{p}^{2}$ Exp
Synthetic	0.27	57.67	0.36	0.02	0.04	0.02
Pietrain	0.35	43.29	0.27	0.02	0.06	0.03
Landrace	0.32	67.61	0.24	0.01	0.05	0.02
Large-White	0.79	37.91	0.33	0.03	0.02	0.01

F(G), frequency of the reference allele G; b, beta coefficient size of the effect; SE, standard error; $\sigma_{a}^{2}$ Exp, genetic variance explained; $\sigma_{p}^{2}$ Exp, phenotypic variance explained.

### Haplotype Analysis

**FIGURE 2:**
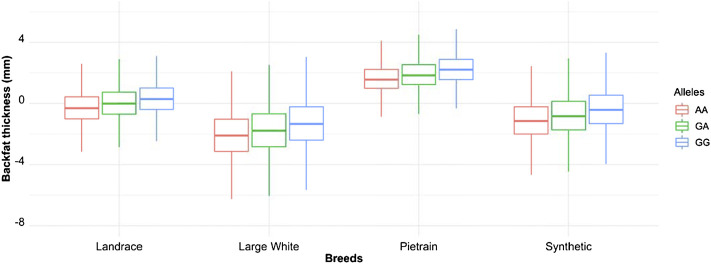
Boxplot showing the additive effect of the rs80985094 G/A allele on backfat among the different populations. Phenotype is backfat thickness in mm.

We phased the genotypes using Beagle 5.1 (Browning and Browning, 2007) and selected the most frequent haplotypes either wich contains the A and G allele in the four populations. Based on the haplotype analyses, we identified a core haplotype with four SNPs surrounding the lead SNP (GC**A**AG) present in all populations (size ∼28 Kb). Assuming that recombination reduced the common interval surrounding a new variant reducing back fat, the region containing the causal mutation was expanded by two more SNPs (one SNP upstream and one SNP downstream) to search for possible functional variants. This increases the final region of interest to 46 kb to search for the causal variant (AGC**A**AGC = Synthetic; AGC**A**AGG = Pietrain; GGC**A**AGA = Landrace; AGC**A**AGG = Large-White). The frequencies of these core haplotypes are 57.70%, 35.87%, 22.67% and 16.85% for each population (Synthetic, Pietrain, Landrace and Large-White, respectively) (Table 5). No common core haplotype surrounding the lead SNP could be identified across the populations surrounding the G allele (Table 6). Hence, we believe that the G to A mutation happened on the GCGAG haplotype and was subsequently selected to produce pigs with less backfat. This underlines the hypothesis that the G allele is the wild-type variant and the A allele marks the derived new variant. In line with this hypothesis, the core haplotype containing the wild type G allele could be identified at low frequency. Linkage disequilibrium (*r* ^2^) between the variants in the core haplotype and the lead SNP (A) range from *r* ^2^ = 0.03 and *r* ^2^ = 0.96 (Table 7). The additive effect of the A and G allele on backfat across the evaluated pig populations is shown in Figure 2.

**TABLE 5:** Most frequent haplotypes decreasing backfat.

Haplotype	N. of haplotypes	Frequency (%)	Population
CCA​TTA​GTT​ACA​GAG​TGA​GCA​AGC​TAT​CGG​GAG​TCG​TGT​GT	35,869	57.70	Synthetic
CCA​TTA​GTT​ACA​GAG​TGA​GCA​AGG​CTA​TCG​GGA​GTC​GTG​TG	11,591	35.87	Pietrain
TCA​AGC​TTG​ACT​CAG​AAG​GCA​AGA​CCT​ATC​GGT​CGT​GTA​CC	17,326	22,67	Landrace
GCC​ATT​ATT​ACA​GAG​TGA​GCA​AGG​ACC​TAT​CGG​GAG​TCG​TG	12,252	16.85	LargeWhite

In red, lead SNP [alternative allele (A)]. In orange, the core region shared by all populations. In green, the neighboring SNPs included to determine the boundaries of the region of interest to 45.7 kb.

**TABLE 6:** Most frequent haplotypes increasing backfat.

Haplotypes	N. of haplotypes	Frequency (%)	Population
CCA​TTA​GTT​ACA​GAG​TGA​GTG​GGC​TAT​AGG​GAG​TCA​CAC​GC	2,948	4.74	Synthetic
TAG​TCG​GGG​GCG​TCA​CAG​ACG​GGA​TCG​CCA​TCG​TCC​ATG​TA	3,599	11.14	Pietrain
TGG​GGC​TGA​GTT​CAG​AAG​ACG​GTA​CCT​ATA​GGT​CAC​ACA​CC	12,692	16,60	Landrace
GCC​ATT​ATT​ACA​GAG​TGA​GTG​GGG​ACC​TAT​AGG​GAG​TCA​CA	17,801	24.48	Large-White

In red, lead SNP [reference allele (G)]. In green, the same region covering the core haplotype as in Table 5.

**TABLE 7:** Linkage disequilibrium (r2) between the lead SNP and the 660K variants present in the core haplotype.

Variant’s position	rs80985094			
	Synthetic	Pietrain	Landrace	Large-white
SSC5:66094630	0.35	0.81	-	0.03
SSC5:66097126	0.33	0.81	-	0.02
SSC5:66100317	0.27	0.17	0.14	0.48
SSC5:66107719	0.51	0.91	0.95	0.23
SSC5:66126122	0.29	0.81	-	0.02
SSC5:66140348	0.38	0.81	0.14	0.04

rs80985094, lead SNP; SSC5, sus scrofa chromosome 5.

### Haplotype-Based Association

A haplotype-based association analysis was performed to compare the association with the lead SNP and the core haplotype (GC**A**AG) as defined in Table 5 (orange haplotype). The association with the core haplotype was lower than the lead SNP (Table 8). This gives further support that the lead SNP is causal.

**TABLE 8:** Most significant SNP (lead SNP—rs80985094) identified in the within-breed association analysis and Haplotype-based association using number of copies of the core haplotype.

Population	Model	-log10 (p-value)	b	SE	$\sigma_{a}^{2}$ Exp	$\sigma_{p}^{2}$ Exp
Synthetic	SNP^a^	57.67	-0.36	0.02	0.04	0.02
	Haplotype^b^	45.92	-0.29	0.02	0.03	0.02
Pietrain	SNP	43.29	-0.27	0.02	0.06	0.03
	Haplotype	40.08	-0.26	0.02	0.05	0.03
Landrace	SNP	67.61	-0.24	0.01	0.05	0.02
	Haplotype	40.68	-0.17	0.01	0.02	0.01
Large-White	SNP	37.91	-0.33	0.03	0.02	0.01
	Haplotype	32.27	-0.30	0.03	0.02	0.01

b, beta coefficient; SE, standard error; $\sigma_{a}^{2}$ Exp, genetic variance explained; $\sigma_{p}^{2}$ Exp, phenotypic variance explained.

a rs80985094, lead SNP.

b GCAAG, core haplotype.

### Whole-Genome Sequence Data Analysis

For fine mapping, we evaluated a total of 659 whole genome sequenced animals, 222 sequenced animals are homozygous for the A allele, 252 animals are heterozygous, and 185 animals are homozygous for the G allele. Analyzing the data of these subset of animals, we found two variants (besides the lead SNP) with high frequency across populations and in high LD with the lead SNP (Table 9).

**TABLE 9:** LD (r2) between the lead SNP SSC5:66103958 and the other variants SSC5:66190273 and SSC5:66097445.

Variants	SSC5:66103958			
	Synthetic	Pietrain	Landrace	Large-white
SSC5:66190273	0.759	0.820	0.882	0.951
SSC5:66097445	0.632	0.781	0.472	0.894

SSC5:66103958: lead SNP; SSC5, sus scrofa chromosome 5

The LD between these variants and the lead SNP varies among the populations (Table 9). The lead SNP and SSC5:66097445 are annotated within the intronic region of the Cyclin D2 (*CCND2*) gene (*ENSSSCG00000038694*), while SSC5:66190273 is annotated in intergenic region upstream of the *CCND2* gene.

### RNA-Seq Analysis in Crossbred Animals Supports *CCND2* as the Causal Gene

We studied the expression of the *CCND2* and neighboring genes in a population of 100 crossbred animals in four different tissues (liver, spleen, lung, muscle). Table 10 shows the association of the lead SNP with the expression of genes in the backfat locus (SSC5:65-67 Mb) in four tissues.

**TABLE 10:** Expression and eQTL results in the SSC5 backfat locus (5:65-67 Mb).

Gene stable ID	Gene name	Gene start (bp)	Gene end (bp)	Liver p-value	Spleen p-value	Lung p-value	Muscle p-value
ENSSSCG00000033544	*NTF3*	65052517	65123788	NotExpr	0.15	NotExpr	0.36
ENSSSCG00000000720	*AKAP3*	65656289	65842142	3.0e-04	1.2e-07	3.9e-09	6.2e-06
ENSSSCG00000000719	*NDUFA9*	65759160	65788646	0.61	0.20	0.08	0.09
ENSSSCG00000000722	*RAD51AP1*	65872281	65897215	0.85	0.15	0.79	0.31
ENSSSCG00000000723	*C12orf4*	65897541	65944185	2.00e-03	0.64	0.70	0.02
ENSSSCG00000032662	*FGF6*	65975838	65990035	NotExpr	NotExpr	NotExpr	0.04
ENSSSCG00000024219	*TIGAR*	66044686	66067377	0.37	0.58	0.47	0.52
ENSSSCG00000038694	*CCND2*	66087379	66114571	3.0e-03	1.6e-05	0.02	0.14
ENSSSCG00000000732	*CRACR2A*	66443491	66661654	NotExpr	0.25	0.51	NotExpr
ENSSSCG00000000734	*TSPAN11*	66852873	66911573	1.00	0.12	0.77	0.92
ENSSSCG00000000735	*TSPAN9*	66913769	67106233	0.69	0.12	0.95	0.08

Table shows the p-value for the eQTL analysis with the lead SNP (5:66103958). Genes not expressed in any of the four tissues (CPM <1) are not shown.

Bp, base pairs; CPM, counts per million; NotExpr, CPM value < 1.

**FIGURE 3:**
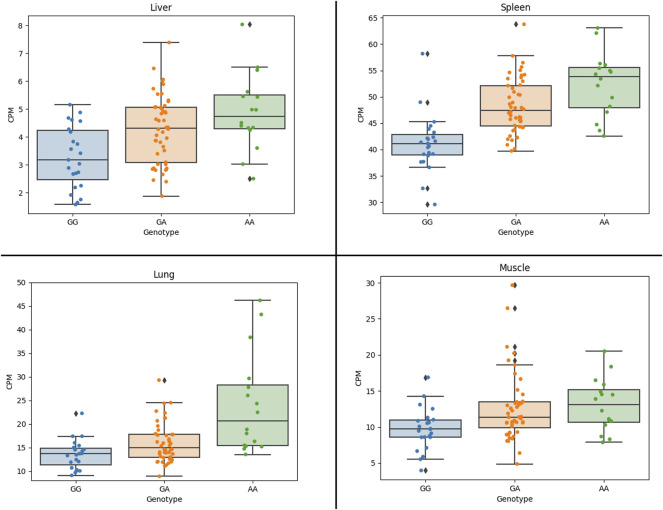
Association of CCND2 expression with the lead SNP in four 87 crossbred individuals (4 tissues). Figure shows CCND2 expression in different genotype classes. G allele is associated with more fat, while the A allele is associated with faster growth. Figure shows that the A allele is significantly associated with increased expression of the CCND2 gene in liver (*p*-value: 3.0 × 10^−3^), spleen (*p*-value: 1.6 × 10^−5^) and lung (*p*-value: 0.02).

The results show that the expression of the *CCND2* gene is significantly associated with the lead SNP in liver, lung and spleen (Figure 3). Also, the expression of the *AKAP3* (all four tissues, although with low expression CPM: 3-5) and the *C12orf4* (liver, muscle) gene show significant association with the lead SNP. The *AKAP3* gene encodes a member of A-kinase anchoring proteins (AKAPs). This protein is highly expressed in spermatozoa may function as a regulator of motility, capacitation, and the acrosome reaction. The *C12orf4* gene encodes a protein involved in mast cell degranulation.

### *CCND2* Regulatory Region

The core haplotype region ranges from SSC5:66094630 to SSC5:66140348 and it covers the *CCND2* gene. Therefore, to discover potentially regulatory elements, we used ChIP-seq and ATAC-Seq public datasets to investigate the region of interest. ChIP-Seq data was used to detect enhancers and promoters marked by H3K4me3, H3K4ac27 and H3K4me1. ATAC-Seq was used to detect open chromatin.

**FIGURE 4:**
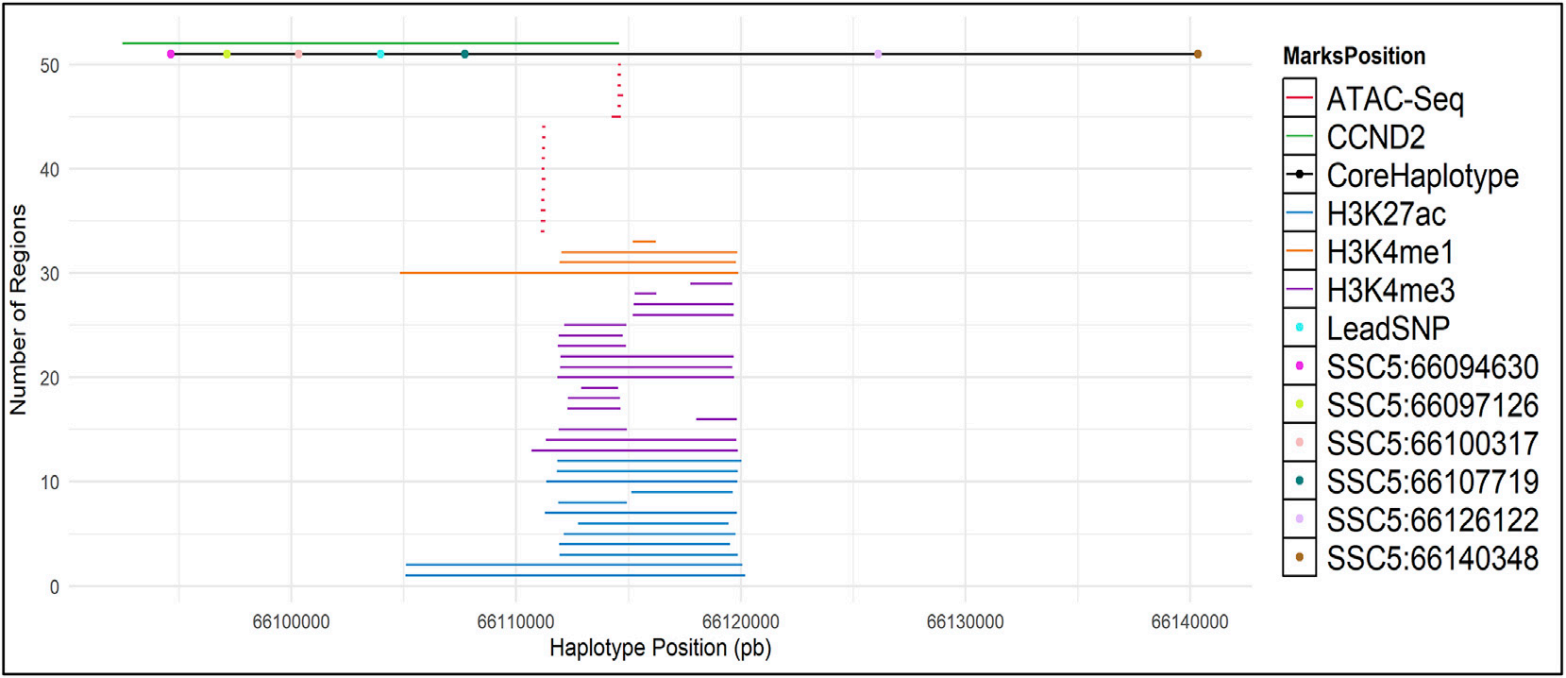
ATAC-Seq and ChIP-Seq (H3K27ac, H3K4me1 and H3K4me3) marks in the CCND2 backfat locus. Broad Peaks in the core haplotype region were called using MACS2.

We have called broad peaks for marks on ATAC-Seq and ChIP-Seq (Figure 4 and Supplementary Material S2). All markers have peak signals just downstream of the lead SNP and are partially covered within the core haplotype region (Supplementary Text S1). In human a predicted promoter is located at the lead SNP location, further supporting a potential regulatory role of the lead SNP (Supplementary Material S3).

## Discussion

Backfat is one of the most important traits in pork production and is closely related to meat quality and efficiency-related traits in pigs. The composition of lipid in backfat is mainly triacylglycerol (Roongsitthichai and Tummaruk, 2014) which exhibits a strong genetic component but is also influenced by environmental factors like feed intake (Wood et al., 1989)In the current study we have confirmed the presence of a QTL region on SSC5 with strong association with backfat in four out of five evaluated pig populations.

The lead SNP (rs80985094) explains up to 6% of the genetic variance and 3% of the phenotypic variance of backfat in the evaluated populations. However, the G allele of the lead SNP is fixed in the Duroc population, associated with increased backfat, explaining the absence of this QTL in Duroc. Durocs are known to have generally higher fat and intramuscular fat content compared to populations of Large White, Landrace, or Pietrain origin.

Several studies have reported QTLs and candidate genes to possibly identify functional mutations in different loci associated with backfat in pigs (Fontanesi et al., 2012; Zhu et al., 2014; Pant et al., 2015). We have identified one strong candidate SNP (rs80985094) located on SSC5:66103958 with very strong association with backfat across populations. Based on the results reported in the PigQTLdb, 19 QTL were previously reported in the same region (SSC5:66 ± 0.5 Mb), in which three of them were related to backfat (QTL identification = 139184, 139225 and 22298) (Onteru et al., 2013; Reyer et al., 2017). According to Reyer et al. (2017), the lead SNP (rs80985094) was also identified as the most significant SNP in the identified region associated with backfat. However, no functional candidate gene was described by these authors for this QTL region. Recently (Delpuech et al., 2021), describe a backfat QTL in the same region in French Large White at rs342862483 (SSC5:66100317) and (Gozalo-Marcilla et al., 2021) pinpoint *FGF23* as a candidate gene in the same QTL region in different pig lines, these differences might be caused due the number of markers used in each study (660K vs 80K). However, given the proximity of the candidate causal SNP and the influence on the expression we argue that the *CCND2* gene is the causal gene affecting backfat at this locus.

Pietrain, Synthetic, and Landrace populations have high frequency for the A allele, which is associated with less backfat, while Large White has a high frequency for the G allele which is associated with more backfat. This result is in line with the selection in the Pietrain, Synthetic, and Landrace populations for leanness and feed efficiency compared to the Large-White population.

The *CCND2* gene was found to be associated with adipose tissue development and differentiation (Vaittinen et al., 2011). Moreover (Le et al., 2017), identified the *CCND2* gene as a possible candidate for backfat conformation in Landrace. These findings are now supported by our results showing that the A allele of the lead SNP is associated with increased expression of the *CCND2* gene in liver, spleen and lung. This finding is in line with previous observations showing that adipogenic triggering leads to a strong downregulation of cell cycle and proliferation genes (including *CCND2*) (Marcon et al., 2019). Moreover, a study in chicken showed that overexpression of *Dnmt3a1* significantly upregulated the mRNA level of cell-cycle-related genes including *CCND2*, but decreasing mRNAs and proteins involved in adipogenesis (Abdalla et al., 2018). In addition, we discovered regulatory elements in the core haplotype region flanking the lead SNP supporting a regulatory role of the causal variant. Together the results reported in literature show that the overexpression of cell-cycle-related genes including the *CCND2* gene suppresses adipogenesis.

In addition to the *CCND2* gene, the core haplotype covers a region also overlaps with the LOC106507534 (a non-conding RNA). In human, the *CCND2* gene region is located on chromosome 12 and LOC106507534 (pig annotation) is orthologous to a long non-coding RNA (lncRNA) named *CCND2-AS1* (human annotation). This lncRNA is associated with regulation of Wnt/b-catenin signaling which directly regulates cell proliferation, cell polarity and cell fate determination during embryo development and tissue homeostasis (Logan and Nusse, 2004; Zhang et al., 2017). Moreover, Zhang et al. (2017) have identified that *CCND2-AS1* plays an important role in glioma cells proliferation through the regulation of the Wnt/b-Catenin pathway. Hence both *CCND2* and *CCND2-AS1* are involved in cell proliferation which is associated with the differentiation of the adipogenic and myogenic cell lineages in early development. However, the pig *CCND2-AS1* was not expressed in our RNA-seq datasets. At the light of these facts, our findings suggest that *CCND2* can also regulate Wnt/b-Catenin pathway in prenatal life impacting fat deposition even after birth in pigs. However, the exact involvement of *CCND2-AS1* with this QTL region remains unclear.

We hypothesize that the lead SNP is the causal variant by affecting *CCND2* gene expression. The association found on SSC5 is highly significant, and the lead SNP explains up to 6% of the genetic variance of backfat in the evaluated populations. The Duroc population was the only population in which we did not identify the QTL. Interestingly, the Duroc population has been selected for improved meat quality and fixation of the G-allele may indicate that this SNP or underlying gene can have some influence in backfat and meat quality. On the other hand, populations highly selected for leanness and feed efficiency (Synthetic, Landrace and Pietrain) show a low frequency of the G allele. If the mutation from G to A occurred in a common ancestor of the white lines, selection will have acted against the G allele in these populations. Due to recombination, the size of the original haplotype on which the mutation occurred is reduced with each generation. Indeed, we still find a core haplotype surrounding the A variant which the four white lines have in common. Furthermore, we also find the original core haplotype with the wild type G allele GC**G**AG at low frequency in all four white populations. Indeed, this haplotype increases back fat in all four lines (Table 11). This further supports the hypothesis that the lead SNP is the causative mutation itself as we cannot identify any other variant in high LD across all four populations in this core region surrounding the A allele. It remains to be elucidated why the G allele is still present at a rather high frequency in commercial pig populations that have been heavily selected for leanness. However, this could be linked to correlated effects on other traits.

**TABLE 11:** Frequency of core haplotype and ancient wild-type core haplotype per line and effects on backfat.

Line	Haplotype	A1	Freq	b	SE	Logpval	$\sigma_{a}^{2}$ Exp	$\sigma_{p}^{2}$ Exp
Synthetic	GCAAG	A	0.7085	-0.29	0.02	45.92	0.029	0.016
	GCGAG	G	0.0636	0.30	0.03	19.12	0.009	0.005
Pietrain	GCAAG	A	0.6437	-0.26	0.02	40.08	0.054	0.027
	GCGAG	G	0.0841	0.20	0.03	8.71	0.011	0.006
Landrace	GCAAG	A	0.6298	-0.17	0.01	42.76	0.025	0.010
	GCGAG	G	0.0001	0.36	0.35	0.52	0.000	0.000
Large White	GCAAG	A	0.2002	-0.30	0.03	32.27	0.017	0.009
	GCGAG	G	0.0181	0.20	0.07	2.59	0.001	0.000

Interestingly, the A allele, associated with less backfat seems to be the ancestral allele in other mammals such as human, horse and cow, where the A allele is present. However, we did not find animals that have the A allele in Asian or European wild boars, indicating that for the pig lineage, the G allele is the ancestral allele. We did however find the A allele in several European local breeds including the Angler Sattelschwein, Bunte Bentheimer, and in Tamworth (Supplementary Material S3). The Angler Sattelschwein is a cross between the German black-and-white Landrace and the Wessex Saddleback, while the Tamworth breed is relatively lean. These results show that the A allele has likely originated in white lines in Europe. However, we cannot exclude recent crossbreeding of European local breeds with breeds that carry the A allele.

We report the *CCND2* gene as a strong candidate gene for backfat deposition in pigs supported by changes in expression and involvement in adipogenesis. However, the exact molecular mechanisms underlying this QTL region remain to be discovered.

## Conclusion

In this study, we identified a QTL region on SSC5 with strong association with backfat across four pig populations. The lead SNP, rs80985094, explained up to 6% of genetic variance of backfat. Gene expression analysis shows that the SNP is associated with the expression of the *CCND2* gene which is involved in adipogenesis during prenatal development and thereby influencing the expressed phenotype in the adult life. Moreover, we believe that the lead SNP is the causal mutation based on fine mapping using the WGS data and the significant eQTL result for the *CCND2* gene. Our results will open up further study to validate the effect of the causal mutation and gene and to better understand its role on fat deposition and muscularity in pigs.

## Data Availability

660K Genotypes, WGS variants (VCF), RNA-sequencing samples, and alignment BAM files are available at the Open Science Framework repository: https://osf.io/xg9cq/.
