# Supplementary material for: Fine Mapping of a Major Backfat QTL Reveals a Causal Regulatory Variant Affecting the CCND2 Gene
Source: Front Genet. 2022 May 25;13:871516. doi: 10.3389/fgene.2022.871516 (PMC9180923; doi:10.3389/fgene.2022.871516)
Supplement: Supplementary file 1 [file Presentation1.zip › Supplementary Material S1.PDF]

Fine mapping of a major backfat QTL reveals a causal regulatory variant affecting the CCND2 gene

Haniel C. Oliveira[1]; Marcos S. Lopes[2,3]; Martijn F. L. Derks[2,4]; Ole Madsen[4]; Barbara Harlizius[2]; Maren van Son[5]; Eli H. Grindflek[5]; Marta Gòdia[4]; Arne B. Gjuvsland[5]; Pamela I. Otto[6]; Martien A. M. Groenen[4]; Simone E. F. Guimaraes[1]

[1]Federal University of Viçosa, Viçosa, Minas Gerais;
[2]Topigs Norsvin Research Center, Beuningen, Netherlands;
[3]Topigs Norsvin, Curitiba, Brazil;
[4]nimal Breeding and Genomics, Wageningen University & Research, Wageningen, the Netherlands;
[5]Norsvin SA, Hamar, Norway
[6]Universidade Federal de Santa Maria, Santa Maria, Brazil

ATAC-Seq (PRJNA665194) and ChIP-Seq (PRJEB28147)

Mapping

bwa-men was used to map the reads against the pig reference genome

```
bwa mem pigReferenceGenome.fa -t 12 00-Fastq/sample1_R1.fastq.gz \
00-Fastq/sample1_R2.fastq.gz > 01-BWA_mapped/sample1_unsort_alig.sam"
```

convert into binary (bam) using samtools view

```
samtools view -@ 12 -bS \
01-BWA_mapped/sample1_unsort_alig.sam > \
01-BWA_mapped/sample1_unsort_alig.bam
```

sort the alignment file

```
samtools sort -@ 12 \
01-BWA_mapped/sample1_unsort_alig.bam > \
01-BWA_mapped/sample1_sort_alig.bam
```

keeping only paired end reads

```
samtools view -f 0x02 -b \
01-BWA_mapped/sample1_sort_alig.bam > \
01-BWA_mapped/sample1_sort_alig_PE.bam
```

remove PCR duplicates

```
samtools rmdup 01-BWA_mapped/sample1_sort_alig_PE.bam \
01-BWA_mapped/sample1_sort_alig.bam_PE_RD.bam
```

Only keep reads that are uniquely aligned against the genome

```
samtools view -@ 12 -b -q 10 \
01-BWA_mapped/sample1_sort_alig.bam_PE_RD.bam > \
01-BWA_mapped/sample1_sort_alig.bam_PE_RD_uniq.bam
```

Indexing the alignment

```
samtools index -@ 12 \
01-BWA_mapped/sample1_sort_alig.bam_PE_RD_uniq.bam
```

Removing MT reads

```
samtools idxstats \
01-BWA_mapped/sample1_sort_alig.bam_PE_RD_uniq.bam | cut -f1 | \
grep -v "MT" | xargs samtools view --threads 6 -b 01-BWA_mapped/ \
sample1_sort_alig.bam_PE_RD_uniq.bam > \
01-BWA_mapped/sample1_sort_alig.bam_PE_RD_uniq_noMT.bam
```

Peak calling

The program MACS2 (https://github.com/taoliu/MACS) is probably the most used program to call ChIP-seq peaks (regions where there is an enrichment of your ChIP-seq mark).

```
macs2 callpeak -f BAMPE -t \
01-BWA_mapped/sample1_sort_alig.bam_PE_RD_uniq_noMT.bam \
-g hs --outdir 02-MACS2_broadPeaks -n sample1_broadPeaks \
-B -q 0.05 --broad --broad-cutoff 0.05
```
