# Supplementary material for: Fine Mapping of a Major Backfat QTL Reveals a Causal Regulatory Variant Affecting the CCND2 Gene
Source: Front Genet. 2022 May 25;13:871516. doi: 10.3389/fgene.2022.871516 (PMC9180923; doi:10.3389/fgene.2022.871516)
Supplement: Supplementary file 1 [file Presentation1.zip › Supplementary Material S2.PDF]

Fine mapping of a major backfat QTL reveals a causal regulatory variant affecting the CCND2 gene

Haniel C. Oliveira[1]; Marcos S. Lopes[2,3]; Martijn F. L. Derks[2,4]; Ole Madsen[4]; Barbara Harlizius[2]; Maren van Son[5]; Eli H. Grindflek[5]; Marta Gòdia[4]; Arne B. Gjuvsland[5]; Pamela I. Otto[6]; Martien A. M. Groenen[4]; Simone E. F. Guimaraes[1]

[1]Federal University of Viçosa, Viçosa, Minas Gerais;
[2]Topigs Norsvin Research Center, Beuningen, Netherlands;
[3]Topigs Norsvin, Curitiba, Brazil;
[4]nimal Breeding and Genomics, Wageningen University & Research, Wageningen, the Netherlands;
[5]Norsvin SA, Hamar, Norway
[6]Universidade Federal de Santa Maria, Santa Maria, Brazil

We have run macs2 software for calling broad peaks from ATAC-Seq and ChIp-Seq data downloaded from ENA database.

Project: PRJNA665194

Gene regulatory elements are central drivers of phenotypic variation and thus of critical importance towards understanding the genetics of complex traits. Here we present a genome-wide annotation of regulatory elements in a non-mammalian vertebrate, chicken (Gallus gallus), as well as two important agricultural mammalian species: pig (Sus scrofa) and cattle (Bos taurus), with chicken and pig in particular being important to human biology and medicine. This report is the first to employ all core assays as defined by the Functional Annotation of Animal Genomes (FAANG) consortium, including information from a wide range of epigenomic assays for the same eight diverse tissues of three livestock species. Comparative analysis of these datasets and those from the human and mouse ENCODE projects revealed that although less than half of enhancers are positionally conserved between species, a core set of regulatory elements are functionally conserved independent of evolutionary distance. Further analysis suggested that tissue-specific transcription factor occupancy at regulatory elements and their predicted target genes were also conserved. Interestingly, the smaller chicken genome – relative to mammals – contains a reduced number of enhancers; however, each chicken enhancer targets more genes, on average, compared to their mammalian counterparts suggesting higher versatility. These datasets and corresponding analysis represent a unique opportunity for the emerging field of comparative epigenomics, as well as animal and human biology and medical research involving species that are globally important food resources. Overall design: ATAC-seq in 8 tissues from 2 livestock species (2 adult male replicates per species). (Text available in the project webpage)

- **Secondary Study Accession:** SRP285093
- **Study Title:** Comparative functional genome annotation of livestock species identifies core amniote regulatory features and avian enhancer versatility (ATAC-Seq)
- **Center Name:** UC Davis
- **Study Name:** Comparative functional genome annotation of livestock species identifies core amniote regulatory features and avian enhancer versatility (ATAC-Seq)

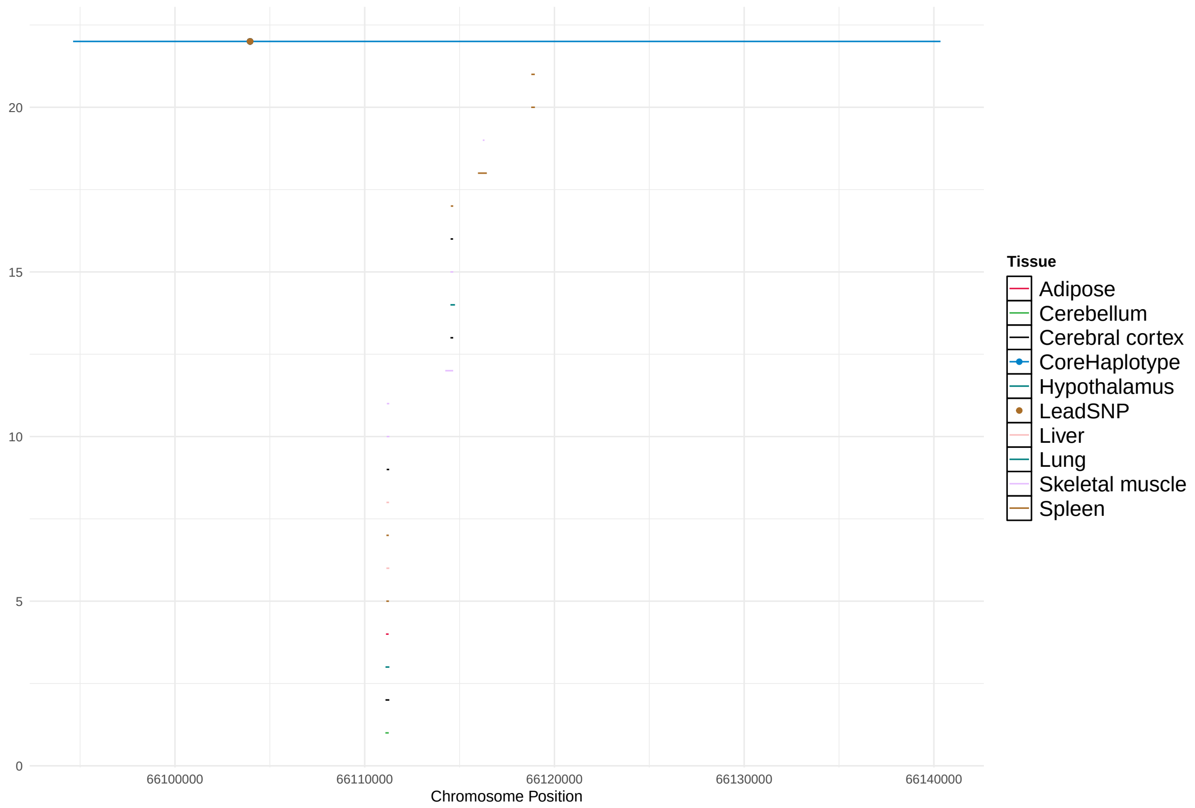

Figure 1: ENA project: PRJNA665194. ATAC-Seq from different tissues. BroadPeaks called using MACS2.

Project: PRJEB28147

To study the tissue-specific evolution of regulatory elements in the mammalian lineage, we created a comprehensive map of promoters and enhancers in 4 tissues of 10 mammalian species. To map in-vivo promoters and enhancers, we performed ChIP-seq experiments for H3K4me3, H3K27ac and H3K4me1 in adult liver, muscle, brain and testis of all 10 species. The species included in the study are: macaque, marmoset, mouse, rat, rabbit, pig, dog, cat, horse and opossum. To correlate regulatory evolution to expression, we also performed RNA-seq experiments in all tissues and species submitted separately to ArrayExpress. (Text available in the project webpage)

- **Secondary Study Accession:** ERP110319
- **Study Title:** H3K4me3, H3K27ac and H3K4me1 ChIP-seq in 4 tissues of 10 mammals
- **Center Name:** Flicek research group European Molecular Biology Laboratory - European Bioinformatics Institute
- **Study Name:** H3K4me3, H3K27ac and H3K4me1 ChIP-seq in 4 tissues of 10 mammals
- **Broker Name:** ArrayExpress

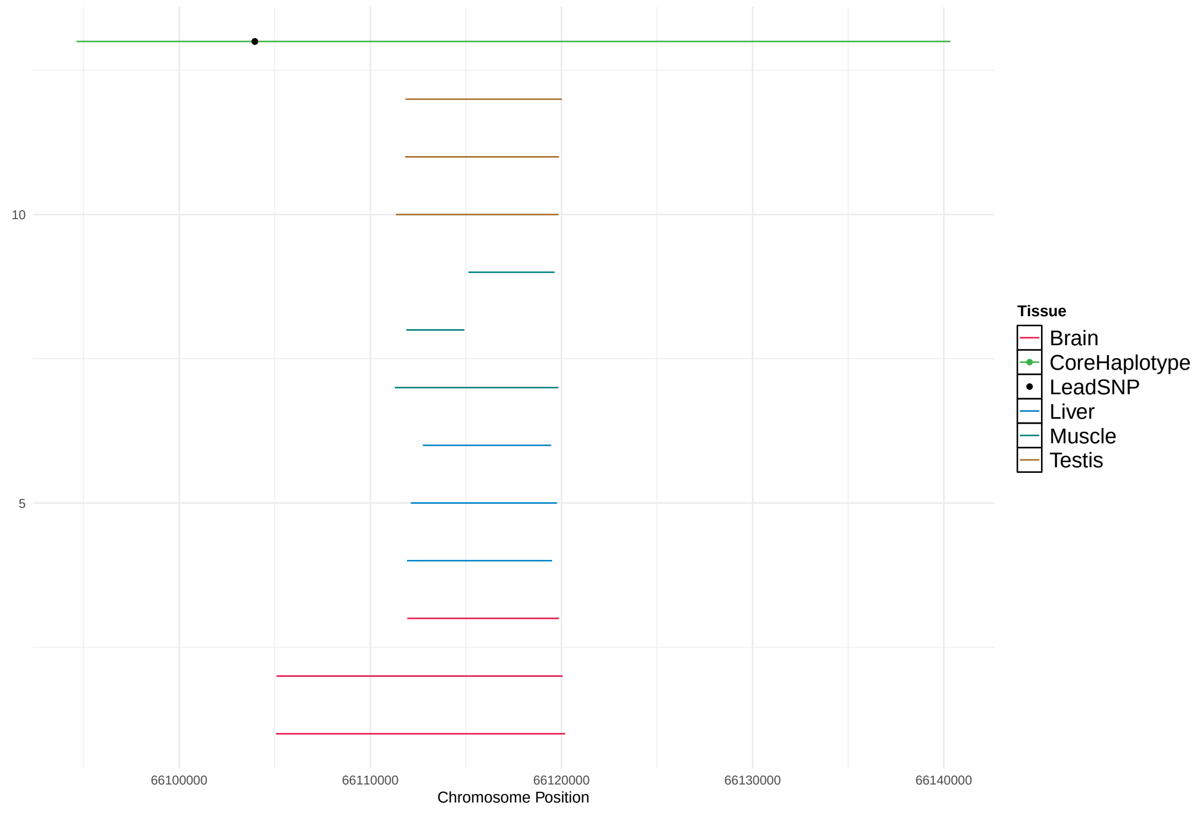

Figure 2: ENA Project: PRJEB28147. H3K27ac Mark in Brain, Liver, Muscle and Testis from pigs. BroadPeaks called using MACS2.

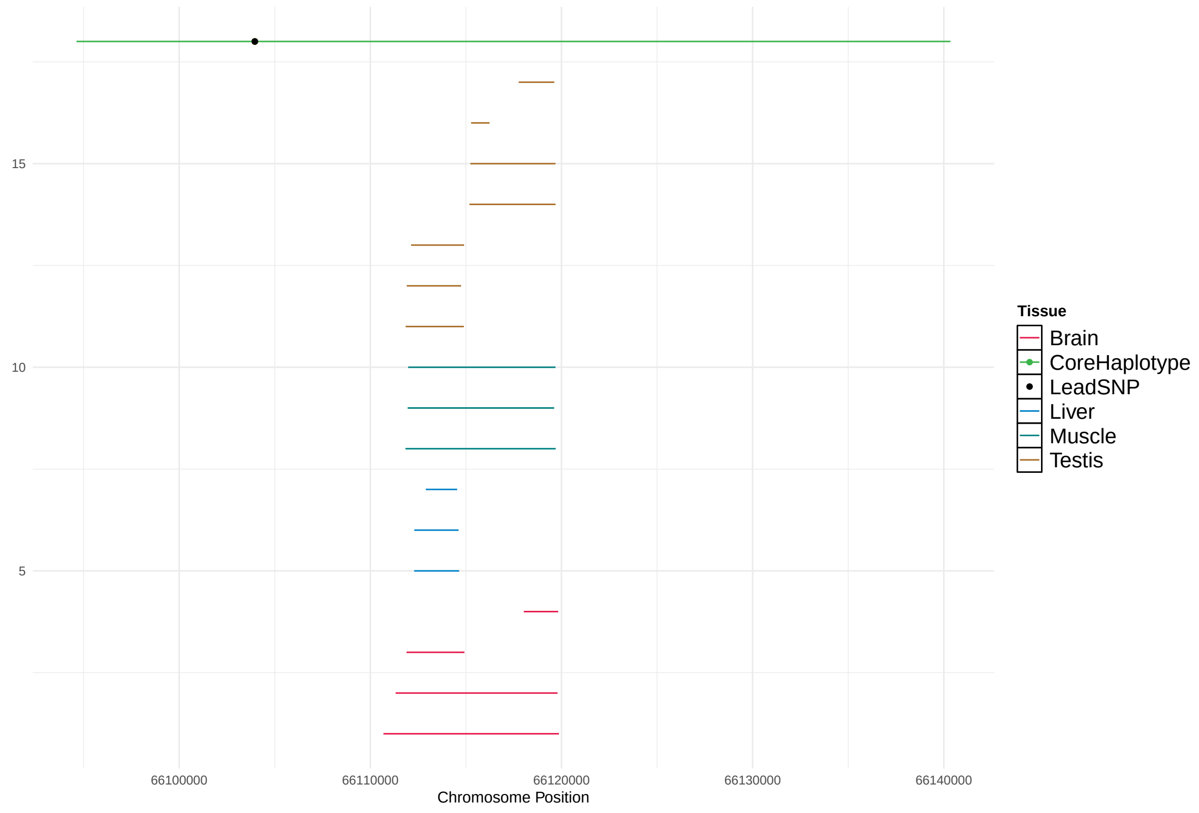

Figure 3: ENA Project: PRJEB28147. H3K4me3 Mark. Brain, Liver, Muscle and Testis. BroadPeaks called using MACS2.

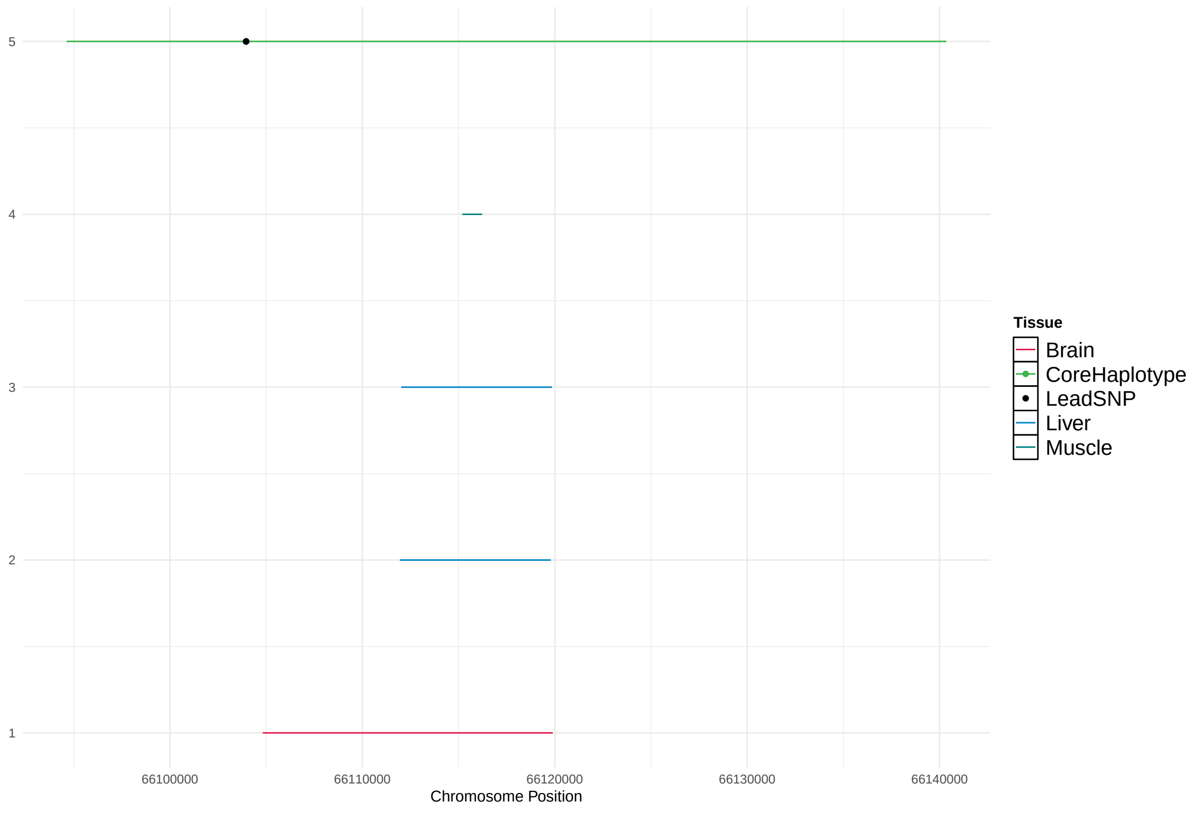

Figure 4: ENA Project: PRJEB28147. H3K4me1 Mark in Brain, Liver, Muscle and Testis. BroadPeaks called using MACS2.

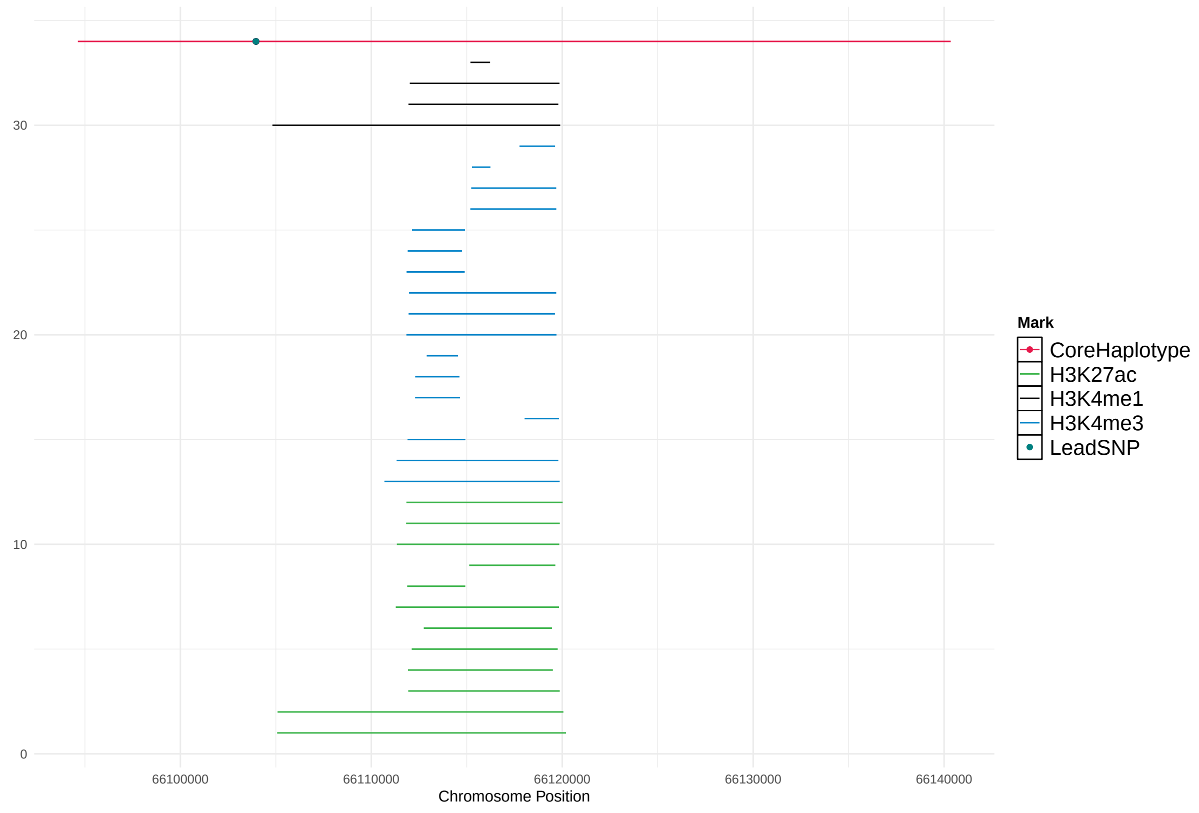

Figure 5: H3K4me3, H3K4me1 and H3K27ac marks in Brain, Liver, Muscle and Testis. BroadPeaks called using MACS2.

Below, the plot for all projects, showing different marks and positions.

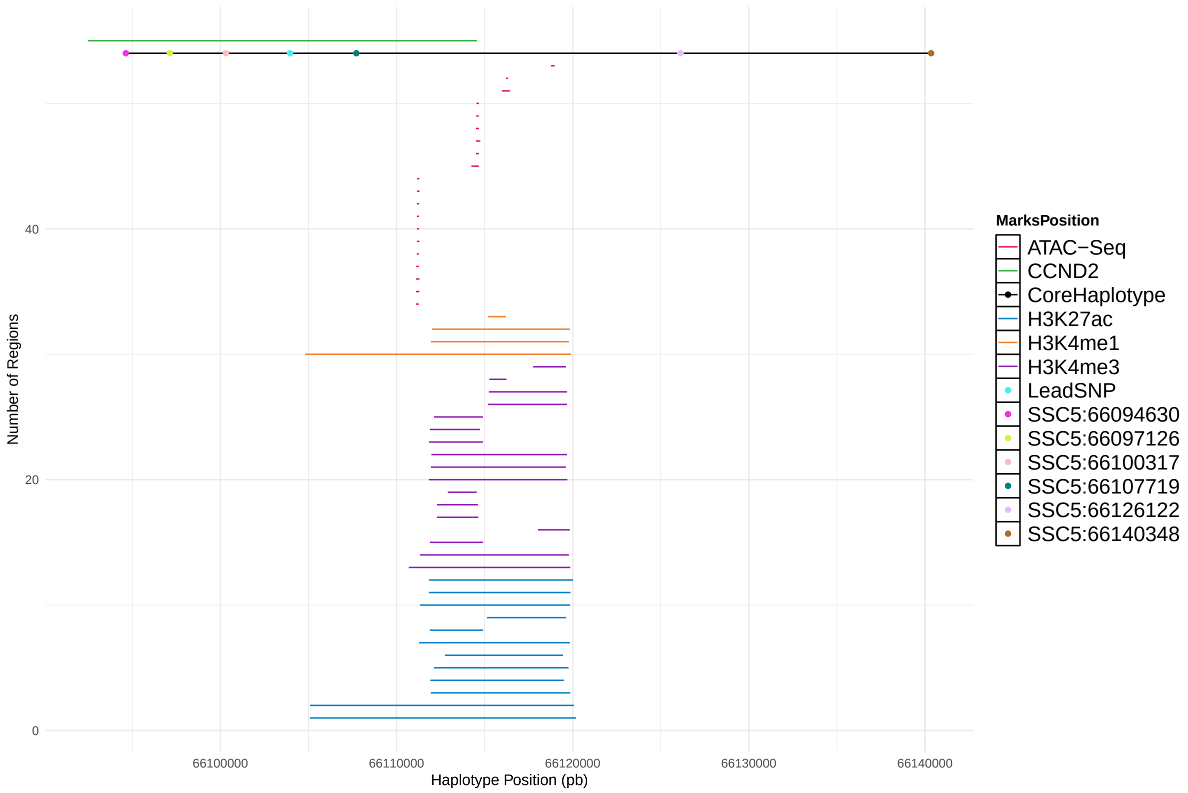

Figure 6: All projects: ATAC-Seq and ChIp-Seq (H3K4me3 and H3K27ac marks) across different tissues from pig. BroadPeaks called using MACS2.
