# Supplementary material for: Fine Mapping of a Major Backfat QTL Reveals a Causal Regulatory Variant Affecting the CCND2 Gene
Source: Front Genet. 2022 May 25;13:871516. doi: 10.3389/fgene.2022.871516 (PMC9180923; doi:10.3389/fgene.2022.871516)
Supplement: Supplementary file 1 [file Presentation1.zip › Supplementary Material S3.PDF]

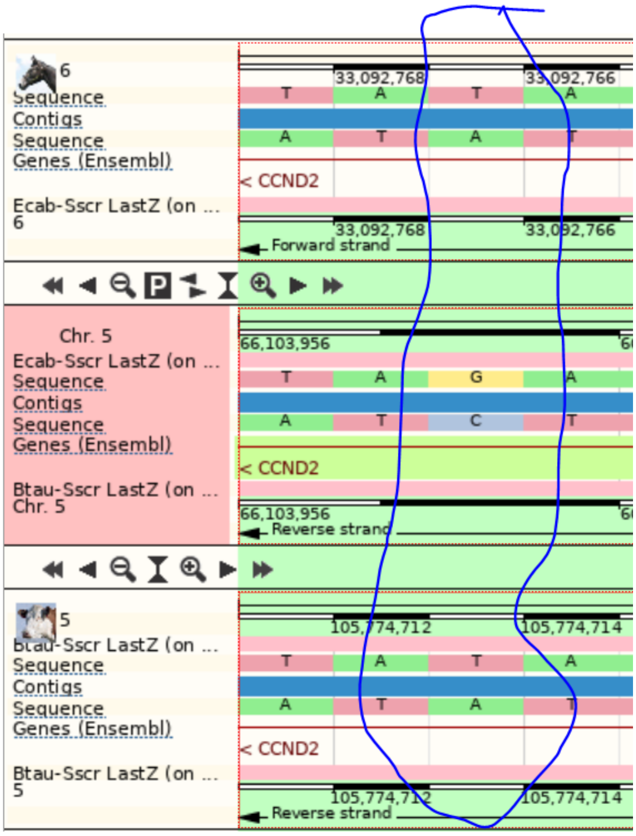

Figure 1: Region of lead SNP in horse and cow. Both species have the A allele at the lead SNP site.

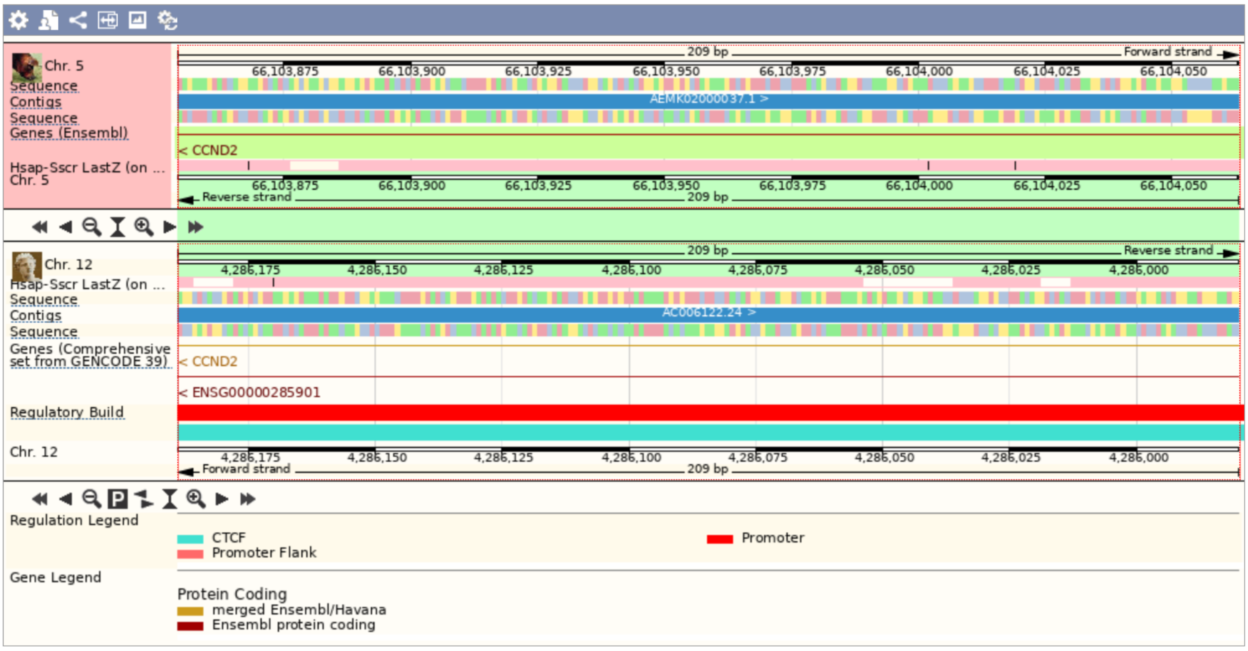

Figure 2: +/- 100 around lead SNP -> promoter and CTCF region in human.

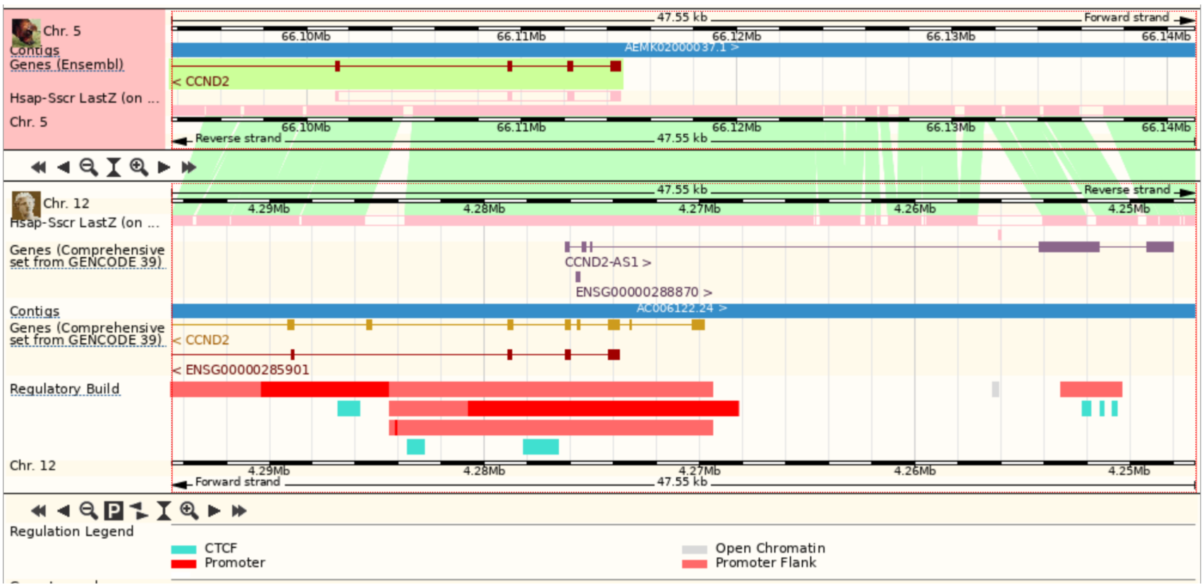

Figure 3: Region covering the core haplotype in pigs and human. Human annotation shows promotor regions in the homologous region.

Table 1: Genotype of the lead SNP in European local breeds.

Sample	Genotype	Breed
AS01F01	0/1	Angler_Sattelschwein
AS01F09	0/1	Angler_Sattelschwein
BB01M47	0/1	Bunte_Bentheimer
BB01M50	0/0	Bunte_Bentheimer
BK01F10	0/0	Berkshire
BK01M20	0/0	Berkshire
BS01F10	0/0	British_Saddleback
BS01F35	0/0	British_Saddleback
CA01F14	0/0	Calabrese
CM01F17	0/1	Chato_Murciano
CM01F18	0/1	Chato_Murciano
CS01F02	0/0	Cinta_Senese
CS01F05	0/0	Cinta_Senese
CT01F01	0/0	Cassertana
CT01F06	0/0	Cassertana
CT01F13	0/0	Cassertana
CT01M12	0/0	Cassertana
CT01M33	0/0	Cassertana
CT01M34	0/0	Cassertana
CT01M35	0/0	Cassertana
GO01F04	0/0	Gloucester_Old_Spot
GO01F23	0/0	Gloucester_Old_Spot
LB01F11	0/0	Large_Black
LB01F49	0/0	Large_Black

LE01F25	0/0	Synthetic
LS01F04	0/0	Linderodsvinn
LS01F09	0/1	Linderodsvinn
MA01F12	0/0	Mangalica
MA01F13	0/0	Mangalica
MA01F18	0/0	Mangalica
MA01F20	0/0	Mangalica
MA01M34	0/0	Mangalica
MA01M35	.	Mangalica
MA01M37	0/0	Mangalica
MW01F29	0/0	Middle_White
MW01F33	0/0	Middle_White
NI01U07	0/0	Negro_Iberico
NI01U28	0/0	Negro_Iberico
NI01U37	.	Negro_Iberico
NS01F05	0/0	Nera_Siciliana
RE01F51	.	Retinto
RE01F54	0/0	Retinto
RE01F62	0/0	Retinto
TA01F19	0/0	Tamworth
TA01M06	1/1	Tamworth
