# Supplementary material for: Fine Mapping of a Major Backfat QTL Reveals a Causal Regulatory Variant Affecting the CCND2 Gene
Source: Front Genet. 2022 May 25;13:871516. doi: 10.3389/fgene.2022.871516 (PMC9180923; doi:10.3389/fgene.2022.871516)
Supplement: Supplementary file 1 [file Presentation1.zip › Supplementary Text S1.PDF]

Fine mapping of a major backfat QTL reveals a causal regulatory variant affecting the CCND2 gene

Haniel C. Oliveira[1]; Marcos S. Lopes[2,3]; Martijn F. L. Derks[2,4]; Ole Madsen[4]; Barbara Harlizius[2]; Maren van Son[5]; Eli H. Grindflek[5]; Marta Gòdia[4]; Arne B. Gjuvsland[5]; Pamela I. Otto[6]; Martien A. M. Groenen[4]; Simone E. F. Guimaraes[1]

[1]Federal University of Viçosa, Viçosa, Minas Gerais;
[2]Topigs Norsvin Research Center, Beuningen, Netherlands;
[3]Topigs Norsvin, Curitiba, Brazil;
[4]nimal Breeding and Genomics, Wageningen University & Research, Wageningen, the Netherlands;
[5]Norsvin SA, Hamar, Norway
[6]Universidade Federal de Santa Maria, Santa Maria, Brazil

The peaks called for ATAC-Seq were in small regions around 5:66111090-66118964 with average length being 181.5 bp, average score 175.2 and log qValue of 17.5. For ChIP-Seq data we have called broad peaks for three marks from four different tissues. For H3K4me3 marks, the peaks were called in regions around SSC5:66110686-66119625 with average of 4.2kb, score and log qValue of 699.2 and 70.0 respectively from brain, liver, muscle and testis. For H3K4ac27, peaks were called in a region around SSC5:66111919-66120021 with average of 8.4 kb, score and log qValue of 333.4 and 33.4 respectively from brain liver, muscle and testis. Peaks of H3K4me1 marks were called in regions around SSC5:66111919-66120021 with average of 7.9 kb, score and log qValue of 154.7 and 15.5 respectively from liver, muscle and testis.
